# Fenómeno techo de cristal en enfermería: revisión integrativa

**DOI:** 10.15649/cuidarte.2261

**Published:** 2022-08-27

**Authors:** Miguel Andrez Valencia-Contrera, Alda Ester Orellana-Yañez

**Affiliations:** 1 Programa Magíster en Enfermería, Universidad de Concepción, Concepción, Chile. Profesor Instructor, Universidad de Antofagasta, Antofagasta, Chile. Email: miguel.valencia@uantof.cl Universidad de Concepción Universidad de Concepción Concepción Chile miguel.valencia@uantof.cl; 2 Universidad de Concepción, Concepción, Chile. Email: aorellan@udec.cl. Universidad de Concepción Universidad de Concepción Concepción Chile aorellan@udec.cl

**Keywords:** Enfermería, Liderazgo, Supervisión de Enfermería, Inequidad de Género, Sexismo, Nursing, Leadership, Nursing, Supervisory, Gender Inequality, Sexism., Enfermagem, Liderança, Supervisão de enfermagem, Iniquidade de Gênero, Sexismo

## Abstract

**Introducción:**

El fenómeno “techo de cristal”, es un término denominado de esta forma por un movimiento feminista de los años ochenta en los Estados Unidos, el cual hace referencia a la barrera “invisible” que impide el progreso de las mujeres en su carrera laboral.

**Objetivo:**

Analizar en el esta- do del arte disponible, la presencia del fenómeno techo de cristal en enfer- mería.

**Materiales y métodos:**

Se realizó una revisión integrativa basada en las cinco etapas de Crossetti, utilizando descriptores (enfermería, consejo directivo, supervisión de enfermería, liderazgo, inequidad de género y se- xismo) validados en el tesauro DeCS y unidas con el booleano AND, en las bases de datos WOS, PUBMED, SCOPUS, SCIELO y BVS.

**Resultados:**

Tras la aplicación de los descriptores y estrategias de búsqueda se localizaron 133 artículos, posterior a la aplicación de filtros se seleccionan 43 artículos, tras aplicar criterios de inclusión y exclusión se obtuvieron 6 artículos, luego se complementó con una búsqueda manual localizando 2 artículos, confor- mándose una muestra de 8 documentos, se aplicó una propuesta de esca- la que asegura la calidad de la muestra, quedando 7 artículos.

**Discusión:**

Todos los artículos seleccionados expresaban una escasa representatividad femenina de enfermería en puestos directivos, se destaca que los hombres son mejor recompensados en términos de remuneración, avance profesio- nal, ocupación de puestos mejores y más prestigiosos.

**Conclusiones:**

Los artículos analizados muestran la presencia del fenómeno techo de cristal, donde las mujeres enfermeras presentan más dificultades que los hombres para acceder a cargos directivos, con una aparente etiología marcada meramente por el género.

## Abstract (en)

**Introduction:**

The glass ceiling effect is a term named after a feminist movement in the 1980s in the United States referring to an “invisible” barrier that impedes women’s progress in their working careers.

**Objective:**

To analyze the presence of the glass ceiling effect in nursing in the available state of the art.

**Materials and Methods:**

An integrative review was conducted following Crossetti’s five steps and using descriptors such as nursing, board of directors, nurs- ing supervision, leadership, gender inequality and sexism, which have been validated in DeCS thesaurus and linked with the Boolean And operation on WoS, PubMed, Scopus, SciELO and BVS databases.

**Results:**

After applying descriptors and search strategies, 133 articles were initially retrieved of which, after applying filters, 43 articles were selected to finally obtain 6 articles after applying inclusion and exclusion criteria. After a manual search, 2 articles were found to make a sample of 8 articles on which a scaling proposal was applied to ensure sample quality, resulting in 7 articles.

**Discussion:**

Selected articles indicated limited female representation of nurses in managerial positions, emphasizing that men are better compensated in terms of remuneration, professional advancement, and better and more prestigious positions.

**Conclusions:**

Analyzed articles reflect the presence of the glass ceiling effect in which female nurses have more difficulties than men in accessing managerial positions by an apparent etiology merely marked by gender.

## Resumen

**Introdução:**

O fenômeno do “teto de vidro”, é um termo que leva o nome de um movimento feminista dos anos 80 nos Estados Unidos, que se refere à barreira “invisível” que impede o pro- gresso das mulheres em suas carreiras profissionais.

**Objetivo:**

Analisar o estado da arte dispo- nível sobre a presença do fenômeno do teto de vidro na enfermagem.

**Materiais e métodos:**

Uma revisão integrativa foi realizada com base nos cinco estágios de Crossetti, utilizando des- critores (enfermagem, diretoria, supervisão de enfermagem, liderança, desigualdade de gênero e sexismo) validados no thesaurus DeCS e ligados ao booleano AND, nas bases de dados WOS, PUBMED, SCOPUS, SCIELO e BVS.

**Resultados:**

Após a aplicação dos descritores e estratégias de pesquisa, foram localizados 133 artigos, após a aplicação dos filtros foram selecionados 43 artigos, após a aplicação dos critérios de inclusão e exclusão foram obtidos 6 artigos, depois uma pesquisa manual foi realizada e foram encontrados 2 artigos, formando uma amostra de 8 documentos, uma escala proposta foi aplicada para garantir a qualidade da amostra, deixando 7 artigos.

**Discussão:**

Todos os artigos selecionados expressaram uma escassa representação fe- minina de enfermagem em cargos de direção, destacando-se que os homens são mais bem re- compensados em termos de remuneração, ascensão profissional, ocupação de cargos melhores e mais prestigiados.

**Conclusões:**

Os artigos analisados mostram a presença do fenômeno do teto de vidro, onde as enfermeiras apresentam mais dificuldades do que os homens no acesso a cargos de gerência, com uma aparente etiologia marcada puramente pelo gênero.

## Introducción

El fenómeno “techo de cristal”, es un término denominado de esta forma por un movimiento feminista de los años ochenta en Estados Unidos, el cual hace referencia al impedimento, limitante o barrera “invisible” que impide el progreso de las mujeres en su carrera laboral^1^.

Con el paso del tiempo el “techo de cristal” se ha hecho notar con más claridad, considerado un vestigio más del lamentable machismo de antaño, vislumbrando una desigualdad meramente marcada por el género. Esto queda demostrado con información proporcionada por la Organización Internacional del Trabajo (OIT), donde describen que la presencia de mujeres en cargos directivos es inferior al de los hombres, e inclusive cuanto más grande es una empresa, menos probabilidades existen que las mujeres accedan a cargos importantes, pese a que las mujeres superan a los hombres en la educación terciaria^2^.

También estudios dan cuenta de la existencia del techo de cristal en diferentes situaciones, tales como: en cinco Universidades chilenas^3^, en empresas mexicanas, donde solo 16 de 500 de las mejores empresas estaban lideradas por mujeres^4^, en empresas brasileñas^5^, en mujeres policías federales de Estados Unidos^6^, o dentro de uno de los mayores grupos hospitalarios franceses^7^; quedando al descubierto copiosos y variados contextos que denotan su presencia, es ahí donde se enmarca su importancia de estudio, más aún cuando es menguada su concientización.

¿Qué se sabe en la actualidad sobre la presencia de dicho fenómeno en enfermería?, la Organización Mundial de la Salud^8^ nos da indicios de un probable resultado, donde refiere en su comunicado del año 2020, que aproximadamente el 90% de todos los profesionales de enfermería son mujeres, pese a ello hay pocas enfermeras ocupando puestos directivos. Bajo esta premisa la presente revisión tiene como objetivo, analizar en el estado del arte disponible, la presencia del fenómeno techo de cristal en enfermería.

## Materiales y Métodos

Investigación secundaria que responde a la metodología de la revisión integrativa, pues proporciona una orientación de síntesis del conocimiento sobre un tema específico, estableciendo de manera transparente criterios que aseguran la calidad de los resultados^9^. Se sigue la metodología de revisión integrativa de Crossetti^10^, la cual consta de cinco etapas:

1. formulación del problema; 2. recolección de datos o definiciones sobre la búsqueda de la literatura; 3. evaluación de los datos; 4. análisis de los datos; y finalmente 5. presentación e interpretación de los resultados.

Dando respuesta a la primera etapa se formuló la pregunta ¿en enfermería los cargos directivos presentan el fenómeno techo de cristal? En la segunda etapa se organizaron las diferentes búsquedas en idioma español, inglés y portugués, utilizando los descriptores DeCS y booleanos descritos en la Tabla 1.

**Tabla 1:** Descriptores y Booleanos utilizados en las búsquedas

	Descriptor en: Español Inglés Portugués	Booleano	Descriptor en: Español Inglés Portugués	Booleano	Descriptor en: Español Inglés Portugués
1		AND	Consejo directive Governing board Conselho director	AND	Inequidad de género Gender inequality Iniquidade de gênero
2	Enfermería Nursing Enfermagem	AND	Consejo directivo Governing board Conselho director	AND	Sexismo Sexism Sexismo
3		AND	Supervisión de enfermería Nursing supervisory Supervisão de enfermagem	AND	Inequidad de género Gender inequality Iniquidade de gênero
4		AND	Supervisión de enfermería Nursing supervisory Supervisão de enfermagem	AND	Sexismo Sexism Sexismo
5		AND	Liderazgo Leadership Liderança	AND	Inequidad de género Gender inequality Iniquidade de gênero
6		AND	Liderazgo Leadership Liderança	AND	Sexismo Sexism Sexismo

Las bases de datos utilizadas son WOS (*Web Of Science*), PUBMED (*United States National Library of Medicine*) con descriptores en inglés, SCOPUS, SCIELO (*Scientific Electronic Library Online*) y BVS (*Biblioteca Virtual en Salud*) en idiomas español, inglés y portugués. Además, se realizó una búsqueda manual consultando el buscador de Google. Las búsquedas fueron realizadas durante el segundo semestre del año 2020. Las estrategias de búsqueda y filtros utilizados en cada base de datos se exponen en la Tabla 2.

**Tabla 2:** Estrategia de búsqueda y filtros aplicados

	Base de datos	Estrategia de búsqueda	Filtros aplicados
1	WOS	Todos los campos (all fields)	-Años de publicación: 2000 - 2020 -Categorías de Web of Science: Nursing -Áreas de investigación: Nursing
2	PUBMED	Todos los campos (all fields)	-Disponibilidad: Texto completo gratis -Fecha de publicación: 20 años -Especies: Humanos -Idioma: español, inglés y portugués
3	SCOPUS	Todos los campos (all fields)	-Año: 2000 - 2020 -Área temática: enfermería -Acceso abierto: todo acceso abierto
4	SCIELO	Todos los índices (all indexes)	-Año de publicación: 2000 - 2020 -Áreas temáticas: ciencias de la salud
5	BVS	Título, resumen, asunto.	-Disponibilidad:texto completo -Año: 2000 - 2020

**Figura 1:**
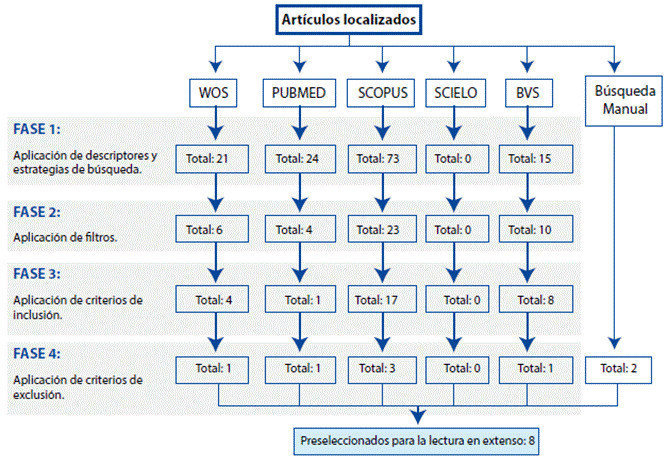
Flujograma de revisión del estado del arte

Los criterios de inclusión utilizados fueron artículos en los tres idiomas mencionados, publicados en el periodo 2000-2020 (hasta el mes de diciembre), que estén relacionados con profesionales de enfermería; los criterios de exclusión corresponden a artículos que no tengan acceso libre, que no den respuesta a la problemática en cuestión, duplicados y cartas del editor. El flujograma de revisión se expone en la Figura 1.

Continuando en la segunda etapa de Crossetti se identificaron tras la aplicación de descriptores y estrategias de búsqueda (FASE 1) 133 artículos en total; posterior a la aplicación de filtros (FASE 2), se localizaron 43 artículos; luego de la aplicación de criterios de inclusión (FASE 3) quedaron 30 artículos; y finalmente tras la aplicación de los criterios de exclusión (FASE 4) quedaron 6 artículos; se consideró complementar con una búsqueda manual para enriquecer los resultados, recuperando 2 artículos que cumplían con los criterios definidos.

Para cumplir con la etapa 3 y 4 de Crossetti, los presentes autores han creado una escala, titulada como “Escala de Evaluación de Artículos con Metodologías Heterogéneas para Revisiones Integrativas” (EAMH), ello en respuesta a la creciente necesidad de poder evaluar los criterios metodológicos mínimos, que deben cumplir los artículos, donde están plasmados los estudios con diferentes diseños de investigación, incluidos en revisiones integrativas, con el fin de resguardar la calidad de la muestra seleccionada. La escala y su puntaje se adjunta en la Tabla 3.

**Tabla 3:** Escala de Evaluación de Artículos con Metodologías Heterogéneas para Revisiones Integrativas (Escala EAMH)

N°	Preguntas evaluadoras	SI	NO	Puntaje	Interpretación del puntaje	
1	¿El artículo define claramente los objetivos?	X		1 punto	0-3 puntos	Artículo no recomendable para el análisis
2	¿El artículo define claramente el tipo de metodología utilizada?	X		1 punto		
3	¿Los objetivos son concordantes con la metodología utilizada?	X		1 punto		
4	¿El artículo justifica la cantidad y tipo de la muestra?	X		1 punto	4-5	Artículo apto para el análisis
5	¿El artículo describe cómo se accedió a la muestra?	X		1 punto		
6	¿Los resultados o conclusiones responden a los objetivos planteados?	X		1 punto	6 puntos	Artículo ideal para el análisis

Se aplicó la escala a los 8 artículos preseleccionados^11^^-^^18^, donde dos artículos obtuvieron un puntaje apto para el análisis^14^^,^^18^, cinco artículos tuvieron un puntaje ideal para el análisis^12^^,^^13^^,^^15^^-^^17^, y uno de los artículos obtuvo 0 puntos^11^, descartándose en los resultados de esta investigación secundaria, puesto que se considera no recomendable para el análisis. La base de datos fue almacena en Mendeley Data^19^.

## Resultados

Finalmente, para dar cumplimiento a la etapa 5 de Crossetti *“presentación e interpretación de los resultados”*, se incorporaron 7 artículos^12^^-^^18^, que pasan a formar la muestra final, ver Tabla 4.

De los 7 artículos, uno de ellos fue identificado en la base de datos PUBMED^12^, tres en SCOPUS^13^^-^^15^, uno en BVS^16^ y los dos restantes se identificaron a través de la búsqueda manual consultando el buscador de Google^17^^-^^18^.

Los autores Casado y Botelo^12^, exponen la representatividad femenina en las sociedades científicas del área de salud, analizada por profesión, ámbito de atención y global, donde las mujeres ocupaban un 22.53% puestos de presidencia, 32.41% puestos directivos y un 36.25% del total de las juntas directivas; concluyendo que en las sociedades enfermeras, farmacéuticas y multiprofesionales la representatividad femenina es mayor a comparación de las sociedades médicas, no obstante, sigue siendo un porcentaje importante de hombres en cargos directivos. En el artículo de Santos RM y colaboradores^13^, se presentan categorías emergentes obtenidas a partir de una revisión, de las cuales *“el ejercicio de la enfermería por parte de los hombres”*, describe cómo el hombre se involucra en el cuidado como una necesidad en relación con la fuerza física, donde se afirma que este atributo masculino denota virilidad, protección y defensa, y puede conferir la máxima clase en relación con sociedad, dependiendo del contexto social, también se asocia con el éxito económico, agresivo, de poder y de conocimiento; otra categoría relevante se denomina *“los hombres en enfermería alcanzan con mayor facilidad posiciones de poder y prestigio debido a su masculinidad”*, describiendo que generalmente los hombres son recompensados con el avance profesional y la ocupación de puestos mejores y más prestigiosos.

Los autores Fedoruk y Pincombe^14^, revelan algunos criterios para el nombramiento del puesto ejecutivo en enfermería en la década de los 90; dentro de estos la aceptación por parte de los grupos dominantes que trabajan en las agencias de atención médica, masculinos y médicos, destacando una escasa participación de enfermería en dichos puestos, ello en respuesta al uso de teorías de gestión diseñadas dentro de un paradigma masculino utilizando el lenguaje de los “hombres”.

En el artículo de Punshon *et al*^15^, los autores describen una ventaja de los hombres en términos de remuneración en comparación con su proporción general en la población de enfermería del Reino Unido. Se indica que en un conjunto de profesionales de enfermería especializados/das, los hombres logran más rápidamente un rol más alto en la banda salarial.

Botello-Hermosa y colaboradores^16^, describen que 20 mujeres ocupan la presidencia de un total de 46 cargos en colegios profesionales de enfermería del Reino Unido, concluyendo que no existe paridad entre hombres y mujeres en el conjunto de los colegios estudiados, pese a que en enfermería la presencia femenina fue mayor que en otros colegios profesionales. Se denota mayor participación de hombres a medida que el nivel de responsabilidad aumenta.

En el artículo de García^17^, se señala que, de 16.573 inscritos en colegios oficiales de enfermería, 10.73% eran hombres y un 89.26% mujeres, de estos el 0,074% de las mujeres y 0,4% de hombres eran directivos, presentando un índice de Techo de Cristal de 5.308, concluyendo que, aunque prevalecen las mujeres en enfermería, es más frecuente ser hombre con cargo directivo.

Los autores Méndez y García^18^, los autores describen que más del 84% de los integrantes de la profesión de enfermería son mujeres y que por ende debería resguardarse la proporción en cargos de poder, tanto en el ámbito de los colegios profesionales como en el de los sindicatos. No obstante, se concluye que la mujer enfermera sigue estando en una posición de inferioridad dentro como fuera de la profesión; dentro, la mujer se ve relegada de los cargos de responsabilidad en colegios profesionales como en el sindicato mayoritario de enfermeras, con porcentajes que no se ajustan a la realidad numérica de las mujeres en la enfermería; fuera de la misma, y en relación con el resto de las profesiones sanitarias, se constata que otras profesiones ocupan de forma mayoritaria los puestos de poder y toma de decisiones.

**Tabla 4:** Estudios seleccionados en la revisión integrativa según autor, año de publicación, país, título, metodología, puntuación de escala EAMH y resultados relevantes

Autor, Año , País	Título	Metodología	Escala EAMH	Resultados relevantes
Casado-Mejía R, Botello-Hermosa A. 2015 España	Representatividad de las mujeres en las sociedades científicas españolas del ámbito de la salud en 2014^12^.	Descriptivo transversal	6 puntos	De 173 sociedades científicas en julio de 2014, 41 tienen presidentas (22,53%). Las mujeres ocupan un 32,41% de puestos ejecutivos y un 36,24% del total de juntas directivas.
Santos RM, Barros LMC, Santos SA, Santos WB, Costa LMC. 2017	La inserción masculina en la Enfermería: ¿qué se ha escrito sobre esta cuestión?^13^.	Revisión integrativa	6 puntos	Los resultados permitieron construir cuatros categorías, de las cuales dos están íntimamente relacionadas con la temática: “el ejercicio de la enfermería por parte de los hombres”, y “los hombres en enfermería alcanzan con mayor facilidad posiciones de poder y prestigio debido a su masculinidad”, describiendo que generalmente los hombres son recompensados con el avance profesional y la ocupación de puestos mejores y más prestigiosos.
Fedoruk M, Pincombe J. 2000 Australia	The nurse executive: Challenges for the 21st century^14^.	Revisión documental	4 puntos	Para que las enfermeras asuman puestos de liderazgo en el sistema de atención de la salud del siglo XXI, las enfermeras líderes tendrán que abandonar los comportamientos y prácticas de gestión tradicionales.
Punshon G, Maclaine K, Trevatt P, Radford M, Shanley O, Leary A. 2019 Reino Unido	Nursing pay by gender distribution in the UK - does the Glass Escalator still exist?^15^.	Descriptive statistics	6 puntos	La ventaja de los hombres en términos de salario es evidente, ya que los hombres están sobrerrepresentados en las bandas superiores en comparación con su proporción general en la población de enfermería del Reino Unido.
Botello-Hermosa A, Casado-Mejía R, Germán-Bes C. 2015 España	Presencia de las mujeres en los órganos de dirección de los colegios profesionales del ámbito de la salid en 2015^16^.	Descriptivo transversal	6 puntos	De 251 colegios profesionales en julio de 2015, 41 (21,91%) la presidencia estaba ocupada por mujeres. También ocupaban el 34,69% de los puestos ejecutivos y el 42,80% del total de las juntas directivas. Los colegios médicos y de enfermería tenían una mujer en la presidencia en el 11,32% y 43,48% respectivamente.
García B. 2018 España	Composición por género de puestos directivos en Enfermería en Castilla y León^17^.	Descriptivo transversal	6 puntos	Aunque prevalecen las mujeres en enfermería, es más frecuente ser hombre con cargo directivo.
Méndez-Salguero A, García-García J. 2019	Escasa presencia de la mujer enfermera en puestos de poder^18^.	Revisión cualitativa y nominal	5 puntos	Se ha demostrado que la enfermería, pese a ser una profesión feminizada, tiene que enfrentarse con el fenómeno del token en su propio mundo, por lo que los hombres dominan las posiciones de poder.

## Discusión

Dentro de los artículos seleccionados^12^^-^^18^ todos expresaban una escaza representatividad femenina de enfermería en puestos directivos; un estudio^12^ describió un 66.67% de cargos ejecutivos ocupados por mujeres enfermeras, cantidad que denota una baja presencia de mujeres en dichos cargos, y no se condice con lo expuesto por la OMS que reconoce que un 90% de los profesionales de enfermería son mujeres, por lo que se debería encontrar una proporción similar.

Se destaca que los hombres son mejor recompensados en términos de: remuneración en comparación con su proporción general en la población de enfermería^15^, con el avance profesional y la ocupación de puestos mejores y más prestigiosos^13^, presentándose el mismo patrón en la presidencia de colegios de enfermería^16^. Esta situación es concordante con lo descrito en otro estudio donde se concluye que la mujer enfermera se ve relegada de los cargos de responsabilidad, tanto en colegios profesionales como de sindicatos, pese a que hay más mujeres que hombres en la profesión^18^. Es relevante destacar la etiología del fenómeno dada por los autores, por un lado, Fedoruk y Pincombe^14^ señalan que los criterios para el nombramiento de un puesto ejecutivo, está desarrollado en base al saber de administradores masculinos y médicos, en respuesta del uso de teorías de gestión en el contexto de un paradigma masculino^20^.

Un elemento interesante de destacar es la exposición de la problemática cuantificada a través del *“Índice Techo de Cristal (ITC)”*, en el contexto de colegios oficiales de enfermería, donde valores de 1 presenta igualdad entre hombres y mujeres, valores sobre 1 se consideran un mayor impedimento para el avance femenino^17^; dicho índice proviene de informes “*She Figures*” de la Unión Europea donde se expone la situación de las mujeres científicas y/o académicas en las universidades y centros de investigación europeos^21^.

## Limitaciones del estudio

Pese a que el presente estudio incorporo grandes bases de datos como: WOS, PUBMED, SCOPUS, SCIELO y BVS para la realización de la revisión, nos limitamos a los artículos publicados en ellas, por lo que consultar otras bases de datos podría enriquecer aún más los resultados. Por otro lado, solo se consideraron artículos en el idioma español, portugués e inglés, por lo que puede ser una limitante, puesto que, al considerar un número mayor de idiomas, aumenta la probabilidad de enriquecer los resultados.

## Conclusiones

A través del presente documento se pudo confirmar una realidad ya introducida por la OMS, donde las mujeres enfermeras tienen más dificultades, barreras o limitantes que los hombres para acceder a cargos directivos, con una aparente etiología marcada meramente por el género. Por tanto, la presente revisión integrativa, responde que efectivamente en enfermería los cargos directivos presentan el fenómeno techo de cristal.

La realidad evidenciada pareciera ser una situación de antaño, un contexto hostil de épocas donde el comportamiento de las mujeres obedecía a un patrón marcado por el machismo, no obstante, es un triste rezago que sorprende con su actual existencia.

Los autores del presente documento manifiestan la necesidad de realizar mediciones a través del índice techo de cristal, ya que, es pertinente tener una visión latinoamericana sobre la problemática abordada, generando una base de datos que proporcione la información suficiente para conocer la magnitud del problema.

Finalmente, se puede agregar que durante la búsqueda en las bases de datos se vislumbró que el fenómeno techo de cristal es una situación no ajena a otras profesiones del área de la salud, siendo probable que sea un fenómeno más frecuente de lo que se tiene conocimiento.
